# The European Human Biomonitoring Initiative (HBM4EU): Human biomonitoring guidance values (HBM-GVs) for the aprotic solvents *N*-methyl-2-pyrrolidone (NMP) and *N*-ethyl-2-pyrrolidone (NEP)

**DOI:** 10.1016/j.ijheh.2021.113856

**Published:** 2021-09

**Authors:** Madlen David, Antje Gerofke, Rosa Lange, Marike Kolossa-Gehring, Petra Apel

**Affiliations:** German Environment Agency (UBA), Corrensplatz 1, 14195 Berlin, Germany

**Keywords:** Aprotic solvents, *N*-ethyl-2-pyrrolidone (NEP), *N*-methyl-2-pyrrolidone (NMP), Human biomonitoring guidance value (HBM-GV), HBM4EU, Risk assessment, 2-HESI, 2-hydroxy-N-ethylsuccinimide, 2-HMSI, 2-hydroxy-N-methylsuccinimide, 5-HNEP, 5-hydroxy-*N*-ethyl-2-pyrrolidone, 5-HNMP, 5-hydroxy-N-methyl-2-pyrrolidone, DNEL, Derived No-Effect Level, ECHA, European Chemicals Agency, EEA, European Environment Agency, GC-EI-MS/MS, gas chromatography-electron ionization-mass spectrometry, GC-MS, gas chromatography-mass spectrometry, *GD*, *gestational day*, HBM, Human Biomonitoring, HBM4EU, European Human Biomonitoring Initiative, HBM-GV, Human Biomonitoring Guidance Value, NEP, N-ethyl-2-pyrrolidone, NMP, N-methyl-2-pyrrolidone, NMSI, N-methylsuccinimide, NOAEC, no observed adverse effect concentration, NOAEL, no observed adverse effect level, POD, point of departure, RAC, Risk Assessment Committee, REACH, Registration, Evaluation and Authorisation of Chemicals, RIVM, National Institute for Public Health and the Environment of the Netherlands, SCCS, Scientific Committee on Consumer Safety, SVHC, Substances of very high concern, TRV, Toxicity Reference Value

## Abstract

Toxicologically and/or epidemiologically derived guidance values referring to the internal exposure of humans are a prerequisite for an easy to use health-based interpretation of human biomonitoring (HBM) results. The European Joint Programme HBM4EU derives such values, named human biomonitoring guidance values (HBM-GVs), for priority substances which could be of regulatory relevance for policy makers and have been identified by experts of the participating countries, ministries, agencies and stakeholders at EU and national level. NMP and NEP are such substances for which unresolved policy relevant issues should be clarified by targeted research. Since widespread exposure of the general population in Germany to NMP and NEP was shown for the age groups 3–17 years and 20–29 years, further investigations on exposure to NMP and NEP in other European countries are warranted. The HBM-GVs derived for both solvents focus on developmental toxicity as decisive endpoint. They amount for the sum of the two specific urinary NMP metabolites 5-HNMP and 2-HMSI and likewise of the two specific urinary NEP metabolites 5-HNEP and 2-HESI to 10 mg/L for children and 15 mg/L for adolescents/adults. The values were determined following a consultation process on the value proposals within HBM4EU. A health-based risk assessment was performed using the newly derived HBM-GV_GenPop_ and exposure data from two recent studies from Germany. The risk assessment revealed that even when considering the combined exposure to both substances by applying the Hazard Index approach, the measured concentrations are below the HBM-GV_GenPop_ in all cases investigated (i.e., children, adolescents and young adults).

## Highlights

• HBM-GVs are a prerequisite for an easy to use health-based risk assessment of human biomonitoring results.
• For NMP and NEP metabolites in urine, respectively, HBM-GVs were set for children and adolescents/adults.
• First HBM exposure data indicate widespread exposure of German children, adolescents and young adults to NMP and NEP.
• The Hazard Index approach revealed that even when combined exposure to both solvents is assessed, HBM-GVs are not exceeded.

## 1 Introduction

Human biomonitoring (HBM) is the most established tool to determine human internal exposure to environmental toxicants in matrices such as blood, urine and hair. To gain further insight into the exposure of the European population to environmental pollutants and to use this information for policy advice, HBM4EU was launched. It is a joint initiative of 30 countries and the European Environment Agency (EEA). One of the overarching goals of HBM4EU is to harmonize and quality assure the tools and methods required for HBM studies, to set priorities for relevant substances to be monitored in humans (Ougier et al., 2021a) and to promote the generation of comparable data within the EU. Health-related human biomonitoring guidance values (HBM-GVs) are key evaluation instruments intended for direct comparison with the measured values. They are defined as the concentration of a substance or its specific metabolite(s) in human matrices at and below which adverse human health effects are, according to current knowledge, not expected. The HBM-GVs for the general population (HBM-GV_GenPop_) are per definition equivalent to the HBM-I values derived by the German HBM Commission (Apel et al., 2017; German HBM Commission, 2007a, German HBM Commission, 2007b; Schulz et al., 2011) and similar to the Biomonitoring Equivalents (BE) introduced by Summit Toxicology (Angerer et al., 2011; Aylward, 2018; Hays et al., 2007, 2008). Since health risks cannot be ruled out if these values are exceeded, this is a ‘strong signal for monitoring the health status of the population, the identification of possible sources of exposure and the assessment of whether and how exposure can be reduced by risk management measures ' (Apel et al., 2020). So far, the concept for the derivation of HBM-GVs and also HBM-GVs for cadmium, selected phthalates and DINCH as well as BPA were derived under HBM4EU (Apel et al., 2020; Lamkarkach et al., 2021; Lange et al., 2021; Ougier et al., 2021b). Further HBM-GVs were derived for BPS and the mycotoxin Deoxynivalenol (DON) and are under preparation for publication.

In the present paper, the derivation of HBM-GVs for NMP (CAS no. 872-50-4) and NEP (CAS no. 2687-91-4) for non-occupationally exposed adults, adolescents and children (HBM-GV_GenPop_) are presented. NMP and NEP are aprotic solvents that are used due to their solvent properties and water solubility in many technical applications and consumer products. In the context of the European chemicals regulation (REACH) both substances are classified as toxic to reproduction (category 1B) and as causing serious eye irritation (ECHA, 2020a, b). NMP is furthermore classified as a skin and respiratory irritant and is on the candidate list for authorisation under REACH. Despite similar effects, NEP is still not included in the EU candidate list. A call for evidence is under preparation for NEP to gather recent information for the preparation of a restriction dossier under Annex XV of REACH. Some uses of NMP, on the other hand, are restricted already under Annex XVII of REACH and consequently NMP shall not be manufactured, or used, as a substance on its own or in mixtures in a concentration equal to or greater than 0.3% after 9 May 2020 unless manufacturers and downstream users take the appropriate risk management measures and provide the appropriate operational conditions to ensure that exposure of workers is below the DNELs (14.4 mg/m^3^ for exposure by inhalation and 4.8 mg/kg/day for dermal exposure). Additionally, NMP and NEP are forbidden in cosmetic products (Annex II, Regulation 1223/2009/EC on Cosmetic Products, as amended by Regulation, 2019/1966/EU, 28 November 2019) and other chemical legislations, e.g. construction product regulation, medical devices directive and different workers regulations are in place. Exposure of the general population to NEP is to be expected through the use of e.g. anti-freezing products, coating products, lubricants and greases, adhesives and sealants, air care products, non-metal-surface treatment products, inks and toners, leather treatment products, polishes, waxes and cleaning products (ECHA, 2020a). For NMP, ECHA has no current data whether or in which consumer products NMP might be used as the restriction of NMP has only recently entered into force (ECHA, 2020b). Dermal and inhalation exposure are the main routes of exposure of the general population to NMP and NEP and might occur especially from spray products, paints, cleaners and toners (Akrill et al., 2002; Anundi et al., 2000; RIVM, 2013a). HBM findings from the population representative German Environmental Survey V (GerES V, 2014–2017) and results from time trend analysis using samples from the German Environmental Specimen Bank (ESB) provide evidence of a wide-spread exposure of the non-occupationally exposed population at least in Germany (Schmied-Tobies et al., 2021; Ulrich et al., 2018). The HBM-GVs for NMP and NEP derived within the framework of the HBM4EU project enable the interpretation of internal exposure data and thus allow a straightforward health-based risk assessment to be carried out. The prerequisite for an appropriate evaluation is that the biomarkers that are used are identical and that the analytical methods are comparable and have been quality assured.

## 2 Methodology for deriving HBM-GVs for NMP and NEP

The general methodology for deriving HBM-GVs for the general population and/or workers, commonly agreed upon under HBM4EU, is described in Apel et al. (2020). It is based on earlier work of the German HBM Commission (German HBM Commission, 1996, German HBM Commission, 2007a, German HBM Commission, 2007b, 2014), Summit Toxicology (Hays et al., 2007, 2008) and the French Agency for Food, Environmental and Occupational Health & Safety (ANSES, 2014) inter alia. Following this concept, NMP and NEP, for which German HBM values have already existed since 2015, were also re-evaluated, taking into account current literature and comments obtained during the consultation. As a result, the HBM Commission's approach for NMP and the selected key study (Saillenfait et al., 2002) was confirmed. In relation to NEP, a slightly different approach was taken with a different key study (Saillenfait et al., 2007 instead of Kaspers et al., 2006), but this resulted in the same numerical values for the HBM-GVs compared to the HBM values. In brief, for the derivation of HBM-GVs for NMP and NEP the available toxicokinetic studies were reviewed to select the most appropriate exposure biomarker(s) for NMP and NEP and to identify their respective molar urinary fraction in percent of the total dose. Furthermore, toxicity studies were reviewed to select critical endpoints for each substance, to identify the relevant key studies and PODs on which the final calculation of the HBM-GVs was based. Assessment factors to extrapolate information from animal studies to humans were applied according to generally accepted rules (ECHA, 2012). Epidemiological studies could not be found in the literature search. Levels of confidence (LoC) were assigned to give an evaluation of the quality and reliability of the data on which the derivation of the HBM-GVs is based. Finally, these newly derived HBM-GVs were used for a health-based risk assessment considering exposure data from two studies from Germany (Schmied-Tobies et al., 2021; Ulrich et al., 2018) for different age groups (children, adolescents and adults).

## 3 Results

### 3.1 Selection of biomarkers of exposure for NMP and NEP

NMP is rapidly metabolized in the liver by gradual oxidation of the pyrrolidone ring. Based on the similar chemical structure of NEP compared to NMP, metabolism is assumed to follow the same routes (Koch et al., 2014). Independent of the exposure route, 5-HNMP (5-hydroxy-N-methyl-2-pyrrolidone, CAS 41194-00-7) and 2-HMSI (2-hydroxy-N-methylsuccinimide, CAS 104612-35-3) for NMP (CAS 872-50-4) and 5-HNEP (5-hydroxy-N-ethyl-2-pyrrolidone, CAS not available) and 2-HESI (2-hydroxy-N-ethylsuccinimide, CAS 63467-80-1) for NEP (CAS 2687-91-4) were identified as the main metabolites both in rats and humans (Åkesson and Jönsson, 1997; Koch et al., 2014; RIVM, 2013a). Experimental animals as well as humans excrete NMP mainly via the kidneys (80% via urine over 24 h in rats and 65% via urine in humans), primarily in form of the abovementioned metabolites and the parent compound (Åkesson and Jönsson, 1997; Sitarek and Kilanowicz, 2006). Regarding the ratios of metabolites in urine, NMP is found in rats and also humans mainly as 5-HNMP (∼70%), and around 30% as 2-HMSI; only around 1–2% consists of NMSI and the parent compound (Åkesson and Jönsson, 1997; Deutsche Forschungsgemeinschaft (DFG), 2008). Half-lives in humans were after inhalation exposure to 40 mg/m³ NMP under resting conditions 3.9 h for NMP, 7.5 h for 5-HNMP and 28 h for 2-HMSI, respectively (Bader et al., 2007). The observed half-lives after oral exposure of humans were 4 h for 5-HNMP, 8 h for NMSI, and 17 h for 2-HMSI (Åkesson and Jönsson, 1997). No glucuronide or sulfate conjugates of the metabolites were identified. Mean urinary excretion fractions (F_UE_) in humans were 44% for 5-HNMP (F_UE_: 0.44) and 20% for 2-HMSI (F_UE_: 0.20). Regarding the elimination half-life of NEP in humans, 7 h for 5-HNEP and 22–27 h for 2-HESI were determined after oral exposure (Koch et al., 2014). After 96 h, the metabolites of NEP were detected in all urine samples analysed and mean urinary excretion fractions were 28.9% for 5-HNEP (F_UE_ (96 h): 0.289) and 21.6% for 2-HESI (F_UE_ (96 h): 0.216). The described main metabolites 5-HNMP and 2-HMSI for NMP as well as 5-HNEP and 2-HESI for NEP are each substance-specific for the parent compound (Deutsche Forschungsgemeinschaft (DFG), 2008; Käfferlein et al., 2013; Meier et al., 2013) and thus suitable as exposure biomarkers. The simultaneous determination of the two main metabolites of NMP and NEP offers the advantage of being able to investigate two biomarkers with different elimination behaviour. While 5-HNMP and 5-HNEP reach their maximum excretion values shortly after the end of exposure and have half-lives of 4–7.5 h (depending on exposure route) and 7 h respectively, 2-HMSI and 2-HESI have half-lives of about 17–28 h and 22–27 h respectively, indicating the accumulation of the substance over several days. Due to the longer elimination, it is to be expected that 2-HMSI and 2-HESI will level short-term peak exposures and thus allow for a more representative exposure estimate (Bader et al., 2007, 2008; Koch et al., 2014) also when measuring spot or morning urine samples, even if 24-h urine samples are indicated as the matrix of first choice when comparing to HBM-GV_GenPop_ (Apel et al., 2020).

Transfer of solvents to human breast milk has been reported (Anderson and Wolff, 2000), but for NMP and NEP further studies are missing. Physico-chemical properties such as molecular weight, lipophilicity and protein binding efficiency are relevant to estimate the possibility for entering in human breast milk. Due to the low log P_ow_ of NMP and NEP and their relatively short half-lives, the accumulation in fat is not very likely and the possibility of a transfer into human milk fat is reduced (Breitzka et al., 1997). But as both NMP and NEP as well as their metabolites have a very small molecular weight and through their solvent properties, it cannot be excluded that they may pass the human membranes into human breast milk via aqueous pores according to their concentration gradient (Kustrin et al., 2002), but at the moment no studies exist to confirm this hypothesis.

Due to the reproductive toxicity and classification as SVHC of NMP and the chemical analogy to its substitute NEP, the German cooperation on human biomonitoring between the Federal Ministry for the Environment, Nature Conservation and Nuclear Safety and the German Chemical Industry Association (VCI) saw the necessity to develop appropriate analytical methods to monitor NMP and its substitute NEP in urine of the general population. The cooperation was established to develop new sensitive analytical methods for those substances in human matrices to which the population may have increased exposure or which are of special toxicological relevance (Kolossa-Gehring et al., 2017). For this purpose, two analytical methods have been developed that consist of a stable isotope dilution analysis using solid phase extraction followed by derivatization (silylation) and GC–EI–MS/MS. The limit of quantification (LoQ) is for both metabolites 5-HNMP and 5-HNEP 2.5 μg/L and for 2-HMSI and 2-HESI each 2 μg/L (Schindler et al., 2012; Ulrich et al., 2018).

### 3.2 Selection of the critical toxicity endpoint for NMP and NEP

The HBM database for both solvents is not sufficient to derive a respective HBM-GV based on the relationship between human internal concentration of the biomarker(s) and related health effects. Furthermore, no toxicologically justified reference value from established bodies (e.g. a TDI value from EFSA) suitable for the derivation of HBM-GVs for the general population is available. Therefore, the derivation of HBM-GVs was based on results of toxicity studies in animals. Critical effects considered relevant for the derivation of HBM-GVs for NMP are effects like the reduced body weight (gain) of dams and offspring, and the embryo/foetotoxic and teratogenic effects observed in studies on reproductive toxicity (RAC and SCOEL, 2016; RIVM, 2013a, b).

Regarding the adverse effects of NEP, ECHA's Risk Assessment Committee (RAC) also highlighted effects on the foetus weight of rabbits after oral exposure and of rats after dermal and oral exposure (ECHA/RAC, 2011). Also, NEP showed effects on post-implantation losses and led to late resorptions in rats after oral exposure. Moreover, teratogenic effects were highlighted in rabbits after dermal and oral and in rats after oral administration of NEP. Skeletal malformations were noted in both species after oral administration, as well as rare cardiovascular malformations in rats after oral administration and in rabbits after dermal and oral administration. For an overview of the relevant studies for the derivation of HBM-GVs see Table 1. It can be concluded that the developmental toxic effects of NEP, especially the incidence of malformations in rats after oral administration are comparable to those of NMP. RAC emphasized that the developmental toxic effects and malformations were specific and not a consequence of maternal toxicity. These effects were regarded as the most critical endpoints for the further derivation of HBM-GVs (ECHA/RAC, 2011).

**Table 1:** Summary of the selected key studies and the supplementary studies of NMP and NEP for the derivation of HBM-GVs.

Substance	Species, strain, sex and number/dose	Route, Duration	Dose (mg/kg bw/d)	NOAEL (mg/kg bw/d)	LOAEL (mg/kg bw/d)	Effects at LOAEL	Reference
NMP	Rat, Sprague-Dawley,	Oral (gavage)	0, 125, 250, 500 or 750	125 (maternal and developmental)	250 (maternal and developmental)	bwg↓ and bw↓ (dams and offspring), external soft-tissue and skeletal malformations of the offspring	Saillenfait et al. (2002)
	21-25 f	GD 6-20					
	**Supplementary study**						
	Rat, Wistar,	Oral (gavage)	0, 150, 450 or 1000	–	150 (lowest dose)	Dams: bw↓ from GD 6, offspring: survival rate↓	Sitarek et al. (2012)
	22-28 f	2 weeks before mating until end of lactation phase, 5 d/w					
NEP	Rat, Sprague-Dawley, 19–24 f	Oral (gavage) GD 6-20	0, 50, 250, 500 or 750	50 (developmental)	250 (developmental)	bwg↓ of foetuses, variations of the skeleton	Saillenfait et al. (2007)
	**Supplementary studies**						
	Rat, Wistar, f/m, 10/10	Inhala-tion,	0, 30, 60, 200 mg/m^3^	60 mg/m^3^ (local) 200 mg/m^3^ (systemic)	200 mg/m^3^ (local)	200 mg/m^3^: degeneration/regeneration of the olfactory epithelium, no systemic effects ≤60 mg/m^3^: no local or systemic effects	(ECHA registration dossier, 2013)
		13 weeks,					
		6 h/d, 5 d/week,					
		“head-nose only”, as vapour					
	Rabbit, Himalayan, 25f	Oral (gavage)	0, 20, 60, 200	60	200 (maternal and developmental)	Dams: bwg↓ and reduced food consumption, relative liver and kidney weights↑; fetuses: skeletal malformations↑	BASF, 2007 (included in ECHA/RAC, 2011)
		GD 6-28					

m: male, f: female, bw: body weight, bwg: body weight gain, GD: gestational day, d: days, h: hours.

### 3.3 Choice of the key studies, PODs, and assessment factors

According to the strategy paper to derive HBM-GVs (Apel et al., 2020) the selection of a key study shall include the exposure pathway that is also considered as the relevant exposure pathway for humans. NMP may enter the human body via the skin, via inhalation or orally and is rapidly distributed in the whole body. Due to the good skin permeability for NMP on the one hand and the low vapour pressure of NMP under standard conditions on the other hand, it can be assumed that the dermal route of exposure is more relevant for the general population than the inhalation route (SCCS, 2011). Whether inhalation exposure occurs as vapour, an aerosol or a mixture of both may also depend on temperature and relative humidity in air (Deutsche Forschungsgemeinschaft (DFG), 1998). Due to the areas of application of NMP, the oral pathway is secondary. However, a conceivable derivation of HBM-GVs on the basis of animal studies with dermal administration is considered too uncertain because of the difficulties in extrapolation, especially with regard to the differences in dermal absorption in rodents and humans. Furthermore, even in the available studies with whole-body inhalation exposure, uncertainties exist regarding the amount of additional dermal and oral intake, so that results from studies with oral exposure were considered more appropriate to derive HBM-GVs. For NMP the oral developmental toxicity study from Saillenfait et al. (2002) on rats was chosen as key study and in addition the study from Sitarek et al. (2012) as supporting study. The oral rat study of Sitarek et al. (2012) refers to reproductive toxicity and shows effects on dams (reduced body weight) and offspring (reduced survival rate) at all doses tested. The study of Saillenfait et al. (2002) resulted in a NOAEL of 125 mg/kg bw/d for maternal and developmental effects, that was only slightly below the 150 mg/kg bw/d that showed already maternal and developmental effects in the study of Sitarek et al. (2012) (see Table 1). Because no NOAEL was identified from the study by Sitarek et al. (2012) and the LOAEL is of the same order of magnitude as the identified NOAEL from the study by Saillenfait et al. (2002), an assessment factor (AF) of 3 was considered appropriate for application to the NOAEL of 125 mg/kg bw/d from the study by Saillenfait et al. (2002) to account for uncertainties in the underlying database, thus leading to a value of 42 mg/kg bw/d. In order to account for inter- and intraspecies differences further AFs of 10 each were applied resulting in a TRV-like value of 0.42 mg/kg bw/d. The TRV (toxicity reference value)-like value is a value for external exposure guidance, calculated according to generally accepted rules, e.g. ECHA (2012) Chapter R.8.

For NEP it can also be assumed that dermal exposure is the most relevant exposure route followed by the inhalation exposure, as already described for NMP. For the reasons given above, the derivation of HBM-GVs dealt with here also considers animal studies with oral exposure, but additionally findings from “head-nose only” inhalation studies documented in the registrant summaries in the registration dossier from ECHA (see Table 1). The selected key study is a developmental toxicity study in rats with a LOAEL of 250 mg/kg bw/d and a NOAEL of 50 mg/kg bw/d (Saillenfait et al., 2007), supported by a further developmental toxicity study in rabbits with a LOAEL of 200 mg/kg bw/d and a NOAEL of 60 mg/kg bw/d (10.13039/100004349BASF, 2007). Application of inter- and intraspecies assessment factors of 10 each resulted in a TRV-like value of 0.5 mg/kg bw/d. Regarding a comparison with the inhalation route of exposure to NEP, the database is not very broad and allows only a rough estimation of the body dose having no effects. A study with subchronic exposure of rats (head-nose only) has been conducted and the summary is available in the registration dossier from ECHA (ECHA registration dossier, 2013). In this study, local effects on the mucous membranes of the nose were observed at the highest applied vapour concentration of 200 mg/m^3^, but no systemic toxic effects were observed. Thus, the highest dose tested in this study, 200 mg/m^3^, is regarded as the NOAEC for systemic effects. Extrapolation of this value regarding study duration and exposure conditions (200 mg/m^3^: 2 × 6/24 × 5/7 = 17.86 mg/m^3^) and extrapolation to body dose per 24 h (17.86 mg/m^3^ x 1.15 (ECHA, 2012) = 20.5 mg/kg bw/d) would lead to a lower dose compared with the NOAEL of the key oral developmental study (50 mg/kg bw/d), but it should be kept in mind that higher concentrations have not been tested so far and we also have no information on a LOAEC for systemic effects via the inhalation path.

### 3.4 Calculation of HBM-GVs for NMP and NEP for the general population

By using the mass balance equation, assuming steady-state conditions (see formula 1), an HBM-GV_GenPop_ for NMP of 15 mg/L for adolescents/adults (rounded value) and 10 mg/L for children (rounded value) is calculated for the sum of the selected urinary exposure biomarkers 5-HNMP und 2-HMSI.

Main urinary excretion fractions used are 44% for 5-HNMP (F_UE_: 0.44) and 20% for 2-HMSI (F_UE_: 0.20) (Åkesson and Jönsson, 1997). As shown by Åkesson and Jönsson (1997) a nearly complete excretion within 24 h can be assumed as parent compound or its metabolites (80% of the dose administered within 24 h). In addition, as the TRV-like value is given in relation to body weight, the amount of urine excreted per day had to be adjusted to the body weight. As estimated by the German HBM Commission and included in the concept paper of HBM4EU (Apel et al., 2020), the daily urine volume adjusted to the body weight is assumed to be 0.02 L/kg bw/d for adolescents and adults (including women of child-bearing age) and 0.03 L/kg bw/d for children.

Formula 1: Mass balance equation for the derivation of an HBM-GV for two exposure biomarkers based on an established TRV or based on a TRV-like value.

(1) $$General\;formula:\frac{\frac{\left[MW\left(Metabolite1\right).Fu(Metabolite2).Fue(Metabolite2)\right]}{MW(Substance)}}{Daily\;urinary\;flow\;rate\;adjusted\;to\;the\;BW}$$

TRV = toxicity reference value; MW = molecular weight; MW (NMP) = 99,1 g/mol; MW (5-HNMP) = 115,1 g/mol; MW (2-HMSI) = 129,1 g/mol; MW (NEP) = 113,2 g/mol; MW (5-HNEP) = 129,2 g/mol; MW (2-HESI) = 143,2; F_UE_ = fractional urinary excretion coefficient; bw = body weight. Average daily urinary flow rates adjusted to bodyweight of 0.03 and 0.02 L/kg bw/d for children and adolescents/adults, respectively.

Regarding NEP, an HBM-GV_GenPop_ of 15 mg/L for adolescents/adults and 10 mg/L for children was calculated for the sum of the selected urinary exposure biomarkers 5-HNEP and 2-HESI. The TRV-like value used for the calculation is 0.5 mg/kg bw/d and the urinary excretion fractions are 28,9% for 5-HNEP (F_UE_ (96 h): 0.289) and 21,6% for 2-HESI (F_UE_ (96 h): 0.216) (Koch et al., 2014). In addition, as the TRV-like value is given in relation to body weight, the daily urinary flow rate had to be adjusted to body weight (0.02 L/kg bw/d for adolescents and adults and 0.03 L/kg bw/d for children).

### 3.5 Level of confidence attributed to the HBM-GV_GenPop_

The information on the level of confidence (LoC) is useful to highlight possible data gaps and to address them to the scientific community. The LoC considers the various uncertainties underlying the derivation of the HBM-GV (e.g. reliability of the key study used to derive the TRV-like value, uncertainties related to the extrapolations leading to the TRV-like value, to toxicokinetic data on the substance of interest, and to the calculation of the final HBM-GV). The overall LoC attributed to the HBM-GV_GenPop_ is set to ‘**medium’** for NMP and “**medium/low**” for NEP. Details regarding single criteria are described in the Appendix. However, a low level of confidence does not necessarily mean a low level of protection, because the HBM-GV derivation is based on very conservative scenarios and default assumptions (Apel et al., 2020).

### 3.6 Risk assessment (RA) based on the newly derived HBM-GV_GenPop_

#### 3.6.1 Comparison of exposure to NMP or NEP with their derived HBM-GV_GenPop_

**Fig. 1:**
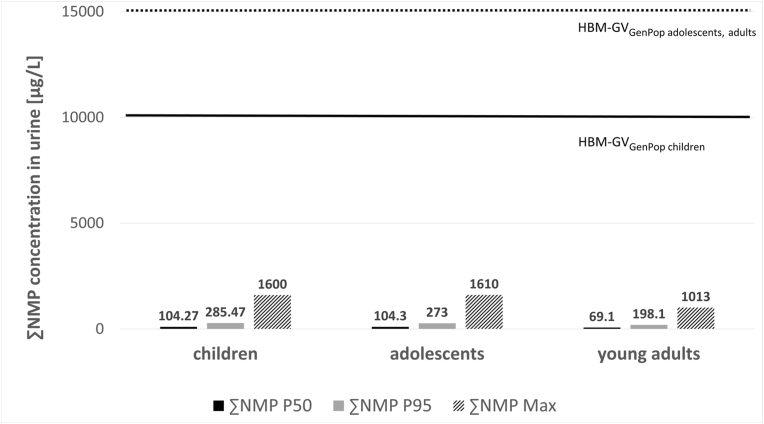
Comparison of exposure data for the sum of NMP metabolites (∑NMP) from GerES V (Schmied-Tobies et al., 2021) and ESB (Ulrich et al., 2018) with the newly derived HBM-GV_GenPop_ values. P50, P95 and maximum values for children aged 3–13 years, adolescents aged 14–17 years and young adults aged 20–29 years for ∑NMP are displayed. The solid black line indicates the HBM-GV_GenPop_ for children whereas the dotted black line indicates the HBM-GV_GenPop_ for adolescents, adults for NMP.

**Fig. 2:**
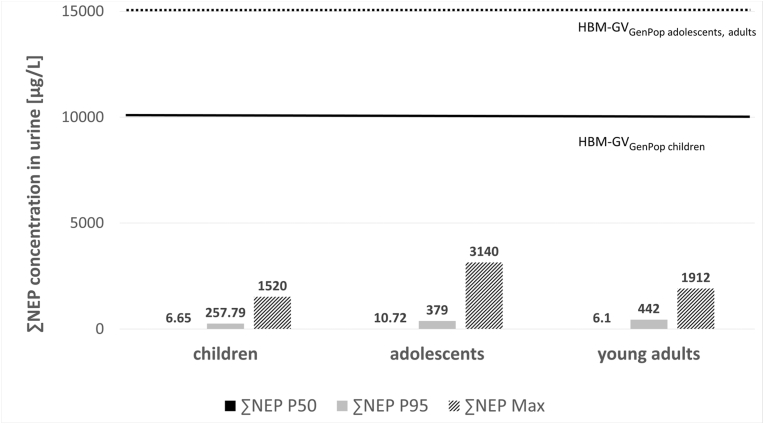
Comparison of exposure data for the sum of NEP metabolites (∑NEP) from GerES V (Schmied-Tobies et al., 2021) and ESB (Ulrich et al., 2018) with the newly derived HBM-GV_GenPop_ values. P50, P95 and maximum values for children aged 3–13 years, adolescents aged 14–17 years and young adults aged 20–29 years for ∑NEP are displayed. The solid black line indicates the HBM-GV_GenPop_ for children whereas the dotted black line indicates the HBM-GV_GenPop_ for adolescents, adults for NEP.

In the following, a risk assessment (RA) is presented which was performed using the exposure data (P50, P95 and maximum values) from two studies from Germany, i.e.: the German Environmental Survey V (GerES V) (Schmied-Tobies et al., 2021) and samples from the German Environmental Specimen Bank (ESB) (Ulrich et al., 2018), and comparing them with the newly derived HBM-GV_GenPop_ for NMP and NEP. Three age groups were considered: children (3–13 years), adolescents (14–17 years) and young adults (20–29 years). For this purpose, the GerES data have been reprocessed to enable the comparison with the respective age groups defined in the derived HBM-GV_GenPop_ for children and the HBM-GV_GenPop_ for adolescents/adults, respectively. The results of this comparison are presented in Fig. 1, Fig. 2.

In all cases considered, even when considering maximum values, the reported exposures (for children and adolescents by Schmied-Tobies et al., 2021; for young adults by Ulrich et al., 2018) are lower than the corresponding HBM-GV_GenPop_ for both NMP and NEP (see Fig. 1, Fig. 2).

The maximum concentration found by Schmied-Tobies et al. (2021) for children from 3 to 13 years, when analysing data from GerES V for ∑NMP was 1600 μg/L (for the age group 11–13 years). This is a factor of 6 below the newly derived HBM-GV_GenPop_ of 10 mg/L for children. The maximum concentration for ∑NEP for children was 1520 μg/L (for the age group 6–10 years) which is a factor of 7 below the HBM-GV_GenPop_ of 10 mg/L for children.

The maximum concentration found by Schmied-Tobies et al. (2021) for adolescents (14–17 years) was 1610 μg/L for the ∑NMP, which is a factor of 9 below the newly derived HBM-GV_GenPop_ of 15 mg/L for adolescents and adults. For ∑NEP the maximum value found for adolescents was 3140 μg/L which is a factor of 5 below the newly derived HBM-GV_GenPop_ for adolescents and adults.

The maximum concentration found by Ulrich et al. (2018) by analysing data from the ESB on students aged 20–29 for ∑NMP was 1013 μg/L. This is a factor of 15 below the newly derived HBM-GV_GenPop_ of 15 mg/L for adolescents and adults. The maximum concentration for ∑NEP was 1912 μg/L which is a factor of 8 below the HBM-GV_GenPop_ of 15 mg/L for adolescents and adults.

#### 3.6.2 Assessing the risk of combined exposure – hazard index (HI) approach

Since in real life a simultaneous exposure to both NMP and NEP is possible and both substances exhibit very similar toxicological profiles, the combined effects of the two substances have to be considered (German HBM Commission, 2015).

In order to assess the cumulative risk of NMP and NEP, the hazard index (HI) approach was applied. Therefore, the HI was calculated as the sum of the hazard quotients (HQs) calculated for NMP and NEP. The HQ for each substance is calculated from the ratio between the sum of 2-HMSI and 5-HNMP concentrations (for NMP) and the sum of 2-HESI and 5-HNEP concentrations (for NEP), respectively, and the corresponding newly derived HBM-GV_GenPop_. A HI < 1 indicates that the exposure is below the newly derived HBM-GV_GenPop_ when considering combined exposure to NMP and NEP (see formula 2).

Formula 2:

(2) $$\frac{Concentration\;in\;urine\;\left(mg\;NMP\;metabolites/L\right)}{HBM-GV_{GenPop}\;\left(mg\;NMP\;metablites/L\;\right)}+\frac{Concentration\;in\;urine\;\left(mg\;NEP\;metabolites/L\right)}{HBM-GV_{GenPop}\;\left(mg\;NEP\;metablites/L\right)}\leq 1$$

In Table 2 the resulting HIs are shown for both studies and for the different age groups investigated, i.e., children (3–13 years), adolescents (14–17 years) and young adults (20–29 years).

**Table 2:** Hazard Index (HI) for NMP and NEP calculated for the respective age groups.

age groups (years of age); study	children (3–13); GerES V	adolescents (14–17); GerES V	adults (20–29); ESB
HI (P50)	0.01	0.01	0.01
HI (P95)	0.05	0.04	0.04
HI (max)	0.3	0.3	0.2

GerES V: German Environmental Survey V (Schmied-Tobies et al., 2021); ESB: German Environmental Specimen Bank (Ulrich et al., 2018).

The HI based on the newly derived HBM-GV_GenPop_ for NMP and NEP when considering co-exposure of these solvents has a maximum of 0.3 [maximum HQs: 0.1 (NMP) and 0.2 (NEP)] using the data from Schmied-Tobies et al. (2021), for children aged 3–13 and adolescents aged 14–17. The calculated HI using the data produced by Ulrich et al. (2018), for young adults aged 20–29 years are in a similar range. The maximum HI was 0.2 [maximum HQs: 0.07 for NMP and 0.1 for NEP] for the young adults.

## 4 Discussion and conclusion

HBM-GVs are an easy to handle health-related risk assessment tool for the interpretation of internal chemical burden. For the general population, HBM-GVs were derived for the sum of the major metabolites of the aprotic solvents NMP and NEP, respectively, which are 10 mg/L for children and 15 mg/L for adolescents/adults. The HBM-GVs have been derived under inclusion of remarks and advice from national experts coordinated via the National Hubs in the HBM4EU partner countries. Expert comments on the first proposal of HBM-GV derivation led to a different choice of key study for NEP, based on the importance of developmental effects compared with grip strength for instance. In the end, however the derived HBM-GVs equal to the HBM-I values from the German HBM Commission, although, as mentioned above, under HBM4EU another key study (Saillenfait et al., 2007) was selected for NEPs value derivation compared to the key study and endpoint/POD (grip strength/BMDL_05_) selected by the German HBM Commission (Kaspers et al., 2006). The Kaspers' study was also used as basis for the derivation of the oral DNEL for systemic chronic effects under REACH. In the case of the DNEL derivation, the overall no observed adverse effect level (NOAEL) of the Kaspers’ study is based on the endpoints body weight and liver effects. To derive the DNEL, an overall assessment factor of 200 was used (ECHA registration dossier, 2020).

One issue that needs further consideration, especially because NMP and NEP are developmental toxicants, are possible differences in human toxicokinetics during pregnancy compared with non-pregnancy. For example, a toxicokinetic study on plasma elimination of NEP after oral exposure of rats showed a much slower transformation in metabolites and also a decrease in metabolites’ elimination rate in pregnant rats compared to non-pregnant rats, also efficient placental transfer of NEP was noted (Bury et al., 2019). This raises the question of whether exposure to NMP and NEP during pregnancy may lead to higher plasma concentration levels and may increase the possibility for health effects. However, there is no information on this from pregnant women to assess the risk. Due to this uncertainty it might be relevant to make use of an additional safety factor when considering women of child-bearing age, but this needs further discussion and agreement.

A literature search revealed that for NMP and NEP, only limited HBM data for the general population are available. HBM data from the German Environmental Specimen Bank show that NMP metabolites 5-HNMP and 2-HMSI could be quantified in 98% and 99.6% of the urine samples and NEP metabolites 5-HNEP and 2-HESI could be quantified in 34.8% and 75.7% of the urine samples of young not occupationally exposed adults. These results clearly indicate that the investigated population is exposed to both NMP and NEP. Median NMP metabolite levels were 30.3 μg/L 5-HNMP as well as 38.8 μg/L 2-HMSI and median NEP metabolite levels were < LOQ for 5-HNEP as well as 6.1 μg/L 2-HESI. 95th percentiles were higher for NEP (5-HNEP: 212 μg/L, 2-HESI: 230 μg/L) than for NMP (5-HNMP: 98.1 μg/L, 2-HMSI: 100 μg/L) (Ulrich et al., 2018).

HBM data for children (3–13 years old, 1530–1557 urine samples) and adolescents (14–17 years old, 634–656 urine samples) from the German Environmental Survey (GerES V) were reprocessed for the respective age groups and compared with the derived HBM-GVs. Results show high frequencies of quantification for NMP (100% for 5-HNMP and 2-HMSI) and NEP (32% for 5-HNEP and 87% for 2-HESI) (Schmied-Tobies et al., 2021). Geometric mean concentrations were 104.0 μg/L (5-HNMP: 56.69 μg/L, 2-HMSI: 45.34 μg/L) for the sum of NMP metabolites for children and 100.7 μg/L (5-HNMP: 54.43 μg/L, 2-HMSI: 44.46 μg/L) for adolescents. For the sum of NEP metabolites geometric mean concentrations of 10.22 μg/L (5-HNEP: 2.71 μg/L, 2-HESI: 6.51 μg/L) for children and 16.97 μg/L (5-HNEP: 4.09 μg/L, 2-HESI: 10.86) for adolescents were determined.

A health-based risk assessment (RA) was performed using the newly derived HBM-GV_GenPop_ and comparing them to exposure data from the two aforementioned studies from Germany for three age groups (i.e., children: 3–13 years, adolescents: 14–17 years and young adults: 20–29 years). The RA revealed that the reported exposure was in all considered cases below the corresponding HBM-GV_GenPop_, even when maximum values were used for this assessment.

Even when the combined exposure to both NMP and NEP was considered by applying the Hazard Index approach, the corresponding HBM-GV_GenPop_ were not exceeded in all considered cases (i.e., children 3–13 years, adolescents 14–17 years, and young adults 20–29 years). Thus, according to current knowledge the exposure reported in the two studies from Germany following a substance-oriented approach does not give an indication of health concern, even when the exposure to both NMP and NEP is considered. Nevertheless, this paper shows widespread exposure of German children, adolescents and young adults to NMP and NEP, as NMP and NEP metabolites could nearly be detected in all investigated samples. It is also important to keep in mind that in real life other reprotoxic substances might also be present, leading to combined exposure which may increase the risk for common effects (Kortenkamp and Faust, 2018). Assessing individual chemicals in isolation, without considering risks associated with combined exposures, is increasingly considered insufficient (Martin et al., 2021). Efforts have already been undertaken to identify pathways that converge at critical nodal points to produce down-stream adverse effects, so called adverse outcome pathways (AOPs) (Kortenkamp, 2020). NMP and NEP should also be included in such considerations to avoid potential underestimation of risk.

As the HBM exposure data from Germany indicate a widespread exposure of the general population to NMP and NEP, it would be of interest to obtain HBM data from other European countries and to evaluate whether country differences regarding possible health risks exist. This would also help to create a more complete picture of exposure to NMP and NEP.

## Funding

The authors received funding from the EU Horizon 2020 Framework Project, HBM4EU, Grant number 733032.

## Declaration of competing interest

The authors declare that they have no known competing financial interests or personal relationships that could have appeared to influence the work reported in this paper.
