# Supplementary material for: The European Human Biomonitoring Initiative (HBM4EU): Human biomonitoring guidance values (HBM-GVs) for the aprotic solvents N-methyl-2-pyrrolidone (NMP) and N-ethyl-2-pyrrolidone (NEP)
Source: Int J Hyg Environ Health. 2021 Sep;238:113856. doi: 10.1016/j.ijheh.2021.113856 (PMC8573589; doi:10.1016/j.ijheh.2021.113856)
Supplement: Multimedia component 1 [file mmc1.docx]

**Appendix**

**Level of confidence (LoC)**

This supplementary material comprises detailed information on the level of confidence (LoC) for each derived HBM-GV for NMP and NEP and their underlying data. An LoC reflects the reliability of the derived HBM-GV (Apel et al., 2020). The LoC considers the various uncertainties underlying the derivation of the HBM-GV (e.g. reliability of the key study used to derive the TRV/TRV-like value, uncertainties related to the extrapolations with regard to the determination of the TRV/TRV-like value, to toxicokinetic data on the substance of interest, and to the calculation of the final HBM-GV). In the following the LoC for each criterion is described and finally summed up to an overall LoC already indicated in the paper.

1. LoC in the nature and quality of the epidemiological and toxicological data

For NMP information on toxicity to humans is very limited. Consequently, the assessment of toxicity is based mainly on animal studies. Several reproductive and developmental toxicity studies on at least two different species via different routes of exposure have been conducted. An overview of toxicity studies on NMP can be found in the registration dossiers of ECHA and also in the dossier of the HBM Commission (ECHA, 2020b; ECHA RAC and SCOEL, 2014; German HBM Commission, 2015a). The toxicokinetic data used for the HBM-GV calculation for NMP is based on one human volunteer study on only 3 males (Åkesson and Jönsson, 1997), thus data on children and females are lacking. In summary, the LoC regarding the nature and quality of the epidemiological and toxicological data on NMP was evaluated as ‘*medium confidence*’.

For NEP information on toxicity to humans is very limited. Therefore, the assessment of toxicity is based mainly on animal studies. Developmental toxicity studies on at least two different species via different routes of exposure have been conducted, repeated-dose toxicity studies concern rats only, which were exposed by oral or inhalation route (ECHA, 2020a). No studies on dermal exposure exist. Chronic or carcinogenicity studies are lacking. Toxicokinetic data for females and children are lacking. Finally, the LoC regarding the nature and quality of the epidemiological and toxicological data for NEP has to be described with ‘*low confidence*’.

1. LoC in the choice of the critical effect and mode of action (MoA)

The evidence of effects on reproduction for NMP is based on several animal studies via different routes of exposure. There is no data giving the indication for a rodent-specific mechanism, thus it can be assumed that these effects are also relevant for humans. However, no evidence for these effects is available from epidemiological studies. Altogether, the LoC in the choice of the critical effect and MoA is stated as ‘*medium confidence*’.

The evidence of the developmental effects of NEP is based on some animal studies via two different routes of exposure (oral and dermal) (ECHA/RAC, 2011; German HBM Commission, 2015b). There is no data giving the indication for a rodent-specific mechanism, thus it can be assumed that these effects are also relevant for humans. However, no evidence for these effects is available from epidemiological studies. Consequently, the LoC in the choice of the critical effect and MoA was assessed as ‘*medium confidence’*.

1. LoC in selected key studies and LoC in critical dose (POD)

For NMP the selected key study is the developmental toxicity study in rats after oral administration of NMP (Saillenfait et al. 2002), supported by the oral rat study of Sitarek et al. (2012) regarding reproductive toxicity. According to ECHA the studies are reliable without restriction and the key study is comparable to a guideline study (OECD Guideline 414 - prenatal developmental toxicity study). Although the main exposure route for NMP is the dermal route, the calculation was based on oral studies. However, a conceivable derivation of HBM-GVs on the basis of animal studies with dermal administration was considered too uncertain because of the difficulties in extrapolation, especially with regard to the differences in dermal absorption in rodents and humans. Furthermore, even in the available studies with whole-body inhalation exposure, uncertainties exist regarding the amount of additional dermal and oral intake, so that results from studies with oral exposure were considered more appropriate to derive HBM-GVs. Consequently, the LoC in the selected key study was evaluated with ‘*medium/high confidence*’.

For NMP, a NOAEL and LOAEL were derived from the oral developmental toxicity study (Saillenfait et al., 2002). Additionally, the oral study of Sitarek et al. (2012) on reproductive toxicity was used. In this study no NOAEL could be determined as effects already occurred at the lowest dose tested (reduced body weight of dams, and reduced survival rates of offspring) (Sitarek et al., 2012). There is only a small margin between the NOAEL of Saillenfait et al. (2002) and the LOAEL of Sitarek et al. (2012). To account for uncertainties in the underlying database an assessment factor of 3 was applied. Therefore, the LoC in critical dose for NMP was set to ‘*medium confidence’*.

For NEP, the selected key study on oral developmental toxicity in rats was assessed by ECHA as reliable with restrictions because the guideline followed is not indicated (Saillenfait et al., 2007). The supporting oral developmental toxicity study in rabbits (ECHA/RAC, 2011) was assessed by ECHA as reliable without restriction. Following same assumptions as for NMP regarding the exposure route, the LoC for the selected key study for NEP was defined as ‘*medium/low confidence*’.

The selected POD for NEP was identified as robust, as different animal studies derived NOAELs in same concentration ranges (50 and 60 mg/kg bw/d) (BASF, 2007; Saillenfait et al., 2007). Consequently, the LoC for the selected POD for NEP was assessed with ‘*medium confidence’*.

1. LoC in inter- and intraspecies extrapolations

For both substances NMP and NEP, assessment factors (AFs) for interspecies and intraspecies variability as well as AFs considering the uncertainty in the database according to default values were used for the calculation of an TRV-like value (ECHA, 2012). Therefore, for both substances the LoC in inter- and intraspecies extrapolations were evaluated with ‘*low confidence’*.

1. Overall LoC for the HBM-GV_GenPop_

Altogether for NMP the global level of confidence in the derived HBM-GVs for the general population is set to ‘*medium confidence’*.

For NEP the overall level of confidence in the derived HBM-GVs for the general population is set to ‘*medium/low confidence*’.

**References**

Åkesson, B., Jönsson, B.A.g., 1997. Major Metabolic Pathway for N-Methyl-2-Pyrrolidone in Humans. Drug Metabolism and Disposition 25, 267-269.

Apel, P., Rousselle, C., Lange, R., Sissoko, F., Kolossa-Gehring, M., Ougier, E., 2020. Human biomonitoring initiative (HBM4EU) - Strategy to derive human biomonitoring guidance values (HBM-GVs) for health risk assessment. International Journal of Hygiene and Environmental Health 230, 113622.

BASF, 2007. N-ethyl-2-pyrrolidone. Prenatal developmental toxicity study in Himalayan rabbits. Oral administration (gavage). Project Number: 40R0033/04058. , included in ECHA RAC (2011) <https://echa.europa.eu/documents/10162/b9c2a03a-b46d-448e-7a52-b8b7f89347eb>.

ECHA, 2012. Guidance on information requirements and chemical safety assessment. Chapter R.8: Characterisation of dose [concentration]-response for human health. Version: 2.1. , <https://echa.europa.eu/documents/10162/13632/information_requirements_r8_en.pdf/e153243a-03f0-44c5-8808-88af66223258>.

ECHA, 2020a. Substance information on 1-ethylpyrrolidin-2-one, <https://echa.europa.eu/de/substance-information/-/substanceinfo/100.018.409> (accessed: 15.08.2020).

ECHA, 2020b. Substance information on 1-methyl-2-pyrrolidone, <https://echa.europa.eu/de/substance-information/-/substanceinfo/100.011.662> (accessed: 04.08.2020).

ECHA RAC and SCOEL, 2014. Opinion on an Annex XV dossier proposing restrictions on 1-Methyl-2-pyrrolidone (NMP), <https://echa.europa.eu/documents/10162/13579/rac_joint_nmp_opinion_en.pdf/e4b4f43b-a3bd-a7c0-08be-16c3886593e7>.

ECHA/RAC, 2011. Annex 1 Background document to the Opinion proposing harmonised classification and labelling at Community level of N-ethyl-2-pyrrolidone (NEP). ECHA/RAC/CLH-O-0000002192-83-01/F, <https://echa.europa.eu/documents/10162/1c8a16a7-d82e-108a-3451-1c826a164a99>.

German HBM Commission, 2015a. Monograph for N-Methyl-pyrrolidone (NMP) and human biomonitoring values for the metabolites 5-Hydroxy-NMP and 2-Hydroxy-N-methylsuccinimide. Bundesgesundheitsblatt Gesundheitsforschung Gesundheitsschutz 58, 1175-1191.

German HBM Commission, 2015b. Stoffmonographie für N-Ethyl-2-pyrrolidon (NEP) und Human-Biomonitoring (HBM)-Werte für die Metaboliten 5-Hydroxy-NEP (5-HNEP) und 2-Hydroxy-N-ethylsuccinimid (2-HESI) im Urin: Stellungnahme der Kommission Human-Biomonitoring des Umweltbundesamtes. Bundesgesundheitsblatt Gesundheitsforschung Gesundheitsschutz 58, 1041-1052.

Saillenfait, A.M., Gallissot, F., Langonné, I., Sabaté, J.P., 2002. Developmental toxicity of N-methyl-2-pyrrolidone administered orally to rats. Food and Chemical Toxicology 40, 1705-1712.

Saillenfait, A.M., Gallissot, F., Sabate, J.P., 2007. Developmental toxic effects of N-ethyl-2-pyrrolidone administered orally to rats. J Appl Toxicol 27, 491-497.

Sitarek, K., Stetkiewicz, J., Wąsowicz, W., 2012. Evaluation of reproductive disorders in female rats exposed to N-methyl-2-pyrrolidone. Birth Defects Res B Dev Reprod Toxicol 95, 195-201.
